# Leaching Characteristics of Potentially Toxic Metals from Tailings at Lujiang Alum Mine, China

**DOI:** 10.3390/ijerph192417063

**Published:** 2022-12-19

**Authors:** Hongyan Zhu, Jinbo Xu, Beibei Zhou, Jia Ren, Qiang Yang, Zhe Wang, Weibo Nie

**Affiliations:** 1 State Key Laboratory of Eco-Hydraulics in Northwest Arid Region, Xi’an University of Technology, Xi’an 710048, China; 2 Shaanxi Mining Development Industry and Trade Corporation Limited, Xi’an 710054, China; 3 Northwest Engineering Corporation Limited Power China, Xi’an 710065, China

**Keywords:** alum mine tailings, potentially toxic metals, soaking test, rainfall leaching, kinetic fitting

## Abstract

To investigate the leaching characteristics and potential environmental effects of potentially toxic metals (PTMs) from alum mine tailings in Lujiang, Anhui Province, soaking tests and simulated rainfall leaching experiments were conducted for two types of slag. PTMs comprising Cd, Cr, Cu, Mn, and Ni were detected in the slag. Cu and Cd contents exceeded the national soil risk screening values (GB 15618-2018). pH values of the two slag soaking solutions were negatively correlated with the solid:liquid ratio. pH values of the sintered slag soaking solutions with different solid:liquid ratios finally stabilized between 4.4 and 4.59, and those of the waste slag soaking solutions finally stabilized between 2.7 and 3.4. The concentrations of Cd, Cr, Cu, Mn, and Ni leached from waste slag were higher than those from sintered slag, and the dissolved concentrations of these PTMs in sintered slag were higher under rainfall leaching conditions than soaking conditions (the difference in Cr concentration was the smallest, 5.6%). The cumulative release of Cd, Cr, Cu, Mn, and Ni increased as the leaching liquid volume increased. The kinetic characteristics of the cumulative release of the five PTMs were best fitted by a double constant equation (R^2^ > 0.98 for all fits). Single factor index evaluations showed that Mn and Ni were the PTMs with high pollution degrees (*P_i_* for Mn and Ni exceed 1) in the leaching solutions. However, considering the biotoxicity of PTMs, the water quality index evaluations showed that the water quality of the sintered slag soaking solution, the waste slag soaking solution, and the sintered slag leachate was good, poor, and undrinkable, respectively. The health risk assessment showed that the total non-carcinogenic risk (HI) values in adults for both the sintered slag leachate and waste slag soaking solution exceeded the safe level of 1, with HI values of 3.965 and 2.342, respectively. The hazard quotient (HQ) for Cd was 1.994 for the sintered slag leachate, and Cd and Cr make up 50.29% and 15.93% of the total risk, respectively. Cr makes up 28.38% of the total risk for the waste slag soaking solution. These results indicate a high non-carcinogenic risk of exposure to Cd and Cr in the leaching solution used for drinking purposes. These findings may provide a reference for the evaluation and ecological control of PTM pollution in alum mining areas.

## 1. Introduction

Due to rapid economic development, the scale of mining is increasing each year. Mining provides energy and resources for humans, but it also causes ecological and environmental problems [1,2,3]. Mining, mineral processing, and smelting processes generate large amounts of waste (including waste rock, main tailings, and smelting slag) [4], which occupy large areas of land and pollute the soil [5], surface water, and groundwater [6,7], as well as affecting plants in the surrounding environment [8] and threatening the health of local people because of the large amounts of potentially toxic metals (PTMs) in waste slag [9,10]. China is a large country with diverse mineral resources. However, due to unreasonable mining, processing, and solid waste treatment, about 800 million tons of mine waste were generated by mining enterprises in China in 2019 [11]. In particular, tailings and wastes are dumped arbitrarily without any treatment in the environment [12], thereby causing severe heavy metal pollution in China.

Mine tailings are recognized as among the main sources of PTMs in the environment [13]. Tailings usually contain Cu, Zn, Hg, Pb, Cd, As, Cr, Mn, Ni, and other PTMs and acidic compounds [14,15], which undergo weathering, oxidation, and rainfall leaching when exposed to the environment, thereby leading to biogeochemical reactions mediated by a combination of air, water, and microbes [16]. Furthermore, acid mine drainage containing various PTMs is constantly released into the surrounding environment [17,18]. PTMs in acid mine drainage migrate with surface runoff or leakage to pollute the surrounding water and soil environments, and they can even threaten human health after entering the body through exposure to the food chain or soil [19], thereby leading to great hidden dangers in the ecological environment in mining areas. Liquids such as rainfall are the key media responsible for the migration of PTMs from tailings [20]. The leaching and release characteristics of PTMs from waste slag directly affect the quality of groundwater and soil [21]. Therefore, understanding the leaching and release patterns of PTMs from tailings is crucial for assessing the risk associated with abandoned tailings and formulating appropriate contamination control strategies.

Many studies have investigated the leaching characteristics of PTMs using static and dynamic leaching tests. Leaching tests can provide important information about the transport of PTMs in tailings [22]. The leaching process by PTMs under environmental conditions can be simulated by using appropriate test conditions and conversion of the solid:liquid ratio and time scale [23]. In particular, standard soil column leaching tests can be performed to understand the long-term release behavior of PTMs under field conditions and to evaluate their environmental impacts [24,25]. Studies have shown that the migration behavior of PTMs depends greatly on the pH and contact time with leaching liquids [26,27]. The amounts of PTMs that leach from tailings are related to the chemical fractions, contents, slag particle size, solid:liquid ratio, and leaching depth [15,25,28,29,30]. In addition, metal sulfides are found in many ores, and acid mine drainage with a low pH will be generated after various reactions that intensify the oxidation and release of minerals and cause the migration of more PTMs into the environment [4].

The Fanshan mining area, located in Lujiang County, Hefei City, Anhui Province, China, has a long history of mining, and it was once an important production base for alum [31]. Alum mines are distinguished from metal and coal mines by the production of waste slag containing alunite (KAl(SO₄)₂·12H₂O) [32]. The ionization of alunite can further aggravate the degree of water acidification caused by pyrite [33]. In the past, alum mining activities produced large amounts of waste slag, some of which comprised low-grade ores discarded from open-pit mining, which accumulated on many parts of the surface without any anti-pollution measures. The other part comprising the waste residue discharged after mineral processing and refining was stacked in and around the tailings pits. The tailings pits accumulated water due to the long-term storage of wastewater discharged from mineral processing, rainfall, and runoff. Open-pit mining produced many depressions in the surface. Under abundant local rainfall, the PTMs in slag exposed to the environment dissolved and migrated with water under rainfall leaching or surface water soaking conditions, thereby causing severe pollution of the Shicao River downstream of the mining area. The Shicao River is one of the main tributaries of the Zhao River. The Zhao River is a significant body of clear water that flows into Chao Lake, which is an important part of the Yangtze River system. The alum mines have been out of production for decades but leaching of the residual waste slag has led to environmental problems in these mining areas, such as extreme acidity and excessive PTM levels in soil and water environments, as well as acid mine drainage (pH of about 3) flowing out of underground mining tunnels all year round, which have severely affected the Zhao River basin and the surrounding ecology. Therefore, it is necessary to study the leaching characteristics of PTMs from alum mine tailings in order to formulate reasonable pollution control and prevention measures.

Previous studies have shown that atmospheric precipitation is the main source of acid mine drainage in the Lujiang alum mining area [34,35]. Water acidification in the alum mining area has two main causes: the hydrolysis of alum in slag is the main cause, and the oxidation of sulfide minerals in slag is the second-most important cause [36,37]. The maximum acid production potential and net acid production potential of alum mine tailings are both high [38]. Large amounts of acid mine drainage are produced in the mining area, and the surrounding environment has been polluted by PTMs to varying degrees. Pollution by PTMs comprising As and Cd is the most severe in the soil in the mining area, and pollution with PTMs comprising Cr, Cu, Mn, Ni, and Zn in the soil varies among different sampling sites [31,37,39]. The source of acid mine drainage containing PTMs from tailings and its impact on the environment surrounding the mining area have been studied previously. However, the leaching and kinetic characteristics of PTMs from alum mine tailings under both soaking and rainfall leaching conditions are still unclear. Therefore, in this study, two types of waste slag from the Lujiang alum mine were investigated in soaking tests and simulated rainfall leaching experiments with the following objectives: (1) to determine the leaching characteristics of PTMs in tailings under the two conditions; (2) to establish kinetic equations for the cumulative release of PTMs under rainfall conditions; and (3) to understand the impacts of PTM leaching and release on the environment to provide a basis for pollution assessments and the ecological management of PTMs in alum mine tailings.

## 2. Materials and Methods

### 2.1. Slag Sampling and Preparation

**Figure 1:**
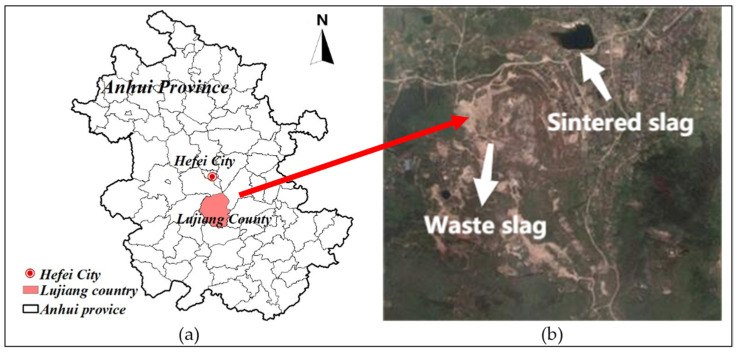
(**a**) Location map showing the study area and (**b**) location where the samples were collected.

The slag samples were collected from Fanshan (southeast of Lujiang County, Anhui Province; Figure 1a). After field investigation, two types of slag were collected, comprising low-grade waste slag stripped from open-pit mining and sintered slag after refining. The sampling location is shown in Figure 1b. For each type of slag, a total of 500 kg of surface slag was collected at 5 points in the same sampling area and then evenly mixed. The collected slag was crushed and passed through a 2 mm sieve for soaking tests and a 10 mm sieve for simulated rainfall leaching experiments.

The main elements in the slag samples are shown in Table 1 and Table S1. The main component of the slag was alum, KAl(SO_4_)_2_·12H_2_O, so the contents of Al and S were high. The Ni and Cr contents in the two types of slag were lower than the risk screening values in the “Soil Environmental Quality and Agricultural Land Soil Pollution Risk Control Standard” (GB 15618-2018) [40], whereas the Cu and Cd contents both exceeded the risk screening values.

### 2.2. Leaching Experiments

#### 2.2.1. Soaking Tests

Five different weights of the two types of slag were weighed, comprising 180 g, 90 g, 36 g, 18 g, and 9 g. The weighed slag samples were placed in polypropylene plastic boxes before 180 g of deionized water was added to each box to prepare solid:liquid ratios of 1:1, 1:2, 1:5, 1:10, and 1:20, respectively. After starting the soaking tests, 50 mL of each supernatant was sampled every day for the first 7 days and every 2 days subsequently. A total of 18 replicate samples were prepared for each test group during a 30 day test period. Thereby, the soaking sample was discarded after the supernatant sample was taken. Destructive sampling was conducted throughout the test. The pH values and heavy metal contents were measured for the supernatant samples.

#### 2.2.2. Simulated Rainfall Leaching Experiments

**Figure 2:**
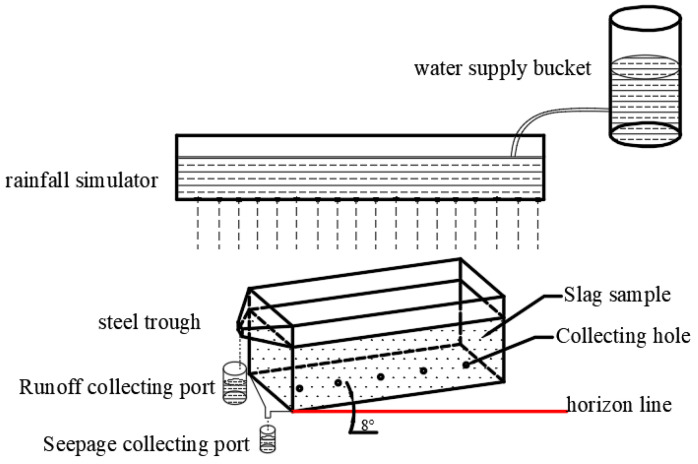
Schematic illustration of a simulated rainfall experimental system.

The experimental system mainly consisted of a pinhole rainfall device measuring 100 cm × 50 cm × 8 cm, a steel trough measuring 100 cm × 20 cm × 40 cm, and a water supply bucket (Figure 2). The steel trough could be set at an inclination angle at any interval of 30° relative to the ground plane. A row of circular holes with a diameter of 1 cm was present in the middle of the bottom, which was used for collecting the leachate. A runoff outlet on the top side was used to collect slope runoff.

The simulated rainfall experiment was designed according to the actual local rainfall in the previous decade. The test lasted for 12 days, and the daily rainfall corresponded to the average monthly rainfall over the previous years. The rainfall intensity was 16 mm/h, and the next rainfall occurred 24 h after the end of the daily rainfall. To ensure that the pH value was close to the actual local weakly acidic rainfall, pure water with a pH of about 6.2 was used as the leaching water. The rainfall distribution and rainfall duration are shown in Table 2.

In this study, the simulated rainfall leaching experiment was only conducted with sintered slag. The slag was loaded into the steel trough to a thickness of 30 cm and compacted every 5 cm. The bulk density was about 2.17 g/cm^3^. Before the experiment, the steel trough was leached with deionized water to saturate the slag. When liquid began to seep through the circular holes at the bottom of the steel trough, the leaching process was stopped, and the experiment was conducted after 48 h. In order to simulate the actual rainfall leaching process for accumulated slag on ground with a certain slope, the angle of inclination between the steel trough and ground was adjusted to 10°. The application of simulated rainfall was stopped when it reached the designated value, and the experiment was finished when no leachate was present at the bottom. During the experiment, the leachate and runoff were collected every hour. The total amount of liquid was recorded, and the pH value and heavy metal contents were measured.

### 2.3. Chemical Analyses and Statistical Analyses

In this study, the main elements were determined in slag samples by X-ray fluorescence spectrometry (XRF-1800; Kyoto, Japan). The sulfur contents of slag samples were determined using a carbon sulfur analyzer (LECO CS230; Saint Joseph, MI, USA). The pH values were determined with a pH meter (PHSJ-5, Shanghai, China). PTMs were analyzed with an inductively coupled plasma-mass spectrometer (iCAP Q ICP-MS; Waltham, MA, USA), which was linearly calibrated from 0 to 1000 μg/L with a multi-element standard solution before running the samples for analysis. All water samples were filtered through a 45 μm membrane and diluted with deionized water. Quality assurance and control for the heavy metal analytical processes were performed by analyzing the Chinese national standard material (GBW07309) and method blanks, and all samples were analyzed in triplicate. The recovery rates were 90.5–114.2%, 96.7–107.7%, 93.1–104.8%, 94.8–103.5%, and 90.2–114.7% for Cd, Cr, Cu, Mn, and Ni, respectively. The detection limits ranged from 0.001 to 0.01 μg/L, depending on the element. All containers were soaked in 1:10 HNO_3_ for 24 h, rinsed with ultrapure water, and dried before use. The reagents used were of analytical grade and high purity. Excel 2021 and Origin 2018 were used for statistical analyses.

### 2.4. Environmental Impact Assessment

The single factor index (*P_i_*) and water quality index (WQI) methods were used to evaluate the impacts of heavy metal leaching from tailings on the water environment. The single factor index (*P_i_*) was calculated as follows:

(1) $$P_{i}=C_{oi}/C_{si}$$

where *P_i_* is the environmental quality index for PTM *i* in water, *C_oi_* is the concentration measured for PTM *i* in water, and *C_si_* denotes the threshold value for PTM *i* in the Chinese Standards for Drinking Water (GB5749-2222) [41]. *P_i_* < 1.0 indicates that the PTM content is below the threshold value for water quality and the water is not polluted. By contrast, the index is higher than the threshold when the PTM pollution degree is high and the water is polluted.

The WQI was calculated as follows:

(2) $$WQI=\sum_{}^{}\left[W_{i}\times \left(\frac{C_{oi}}{C_{si}}\right)\right]\times 100$$

where *W_i_* is the relative weight for a single PTM type calculated as: $W_{i}=w_{i}/\sum w_{i}$, where *w_i_* is the attributed weight for PTM *i* in drinking water according to its relative perceived effects on human health and significance for drinking purposes. The weight values for different PTMs were as described by Xiao et al. in 2019 [42]. According to the WQI values obtained, the water quality can be classified into five categories: excellent (WQI < 50), good (50 ≤ WQI < 100), poor (100 ≤ WQI < 200), very poor (200 ≤ WQI < 300), and undrinkable (WQI > 300) [25,42].

The health risk posed by exposure to the PTMs comprising Cd, Cr, Cu, Mn, and Ni in the leaching solution through drinking water ingestion was quantified using the hazard quotient (HQ) method of the USEPA (1999) [43]:

(3) $$HQ_{i}=CDI_{i}/R_{f}D,$$

(4) $$CDI_{i}=\frac{C_{i}\times IR}{BW},$$

where *CDI* is the dose of metal intake (mg/kg/day) and *R_f_D* is the reference dose (mg/kg/day), which refers to the maximum acceptable dose of a toxic substance. The *R_f_D* values of Cd, Cr, Cu, Mn, and Ni for exposure through drinking water ingestion were 0.0005, 0.003, 0.037, 0.14, and 0.02 mg/kg/day, respectively [44,45]. *IR* is the ingestion rate of water, which was set to 3.49 L/day for adults [45], and *BW* is body weight, which was taken as 70 kg for adults [46].

The hazard index (HI) reflects the cumulative non-carcinogenic risk of multiple pollutants. The HI can be calculated from the following equation [45]:

(5) $$HI=\sum_{i=1}^{n}HQ_{i}$$

an *HQ* or *HI* value greater than 1 indicates a high chronic health risk [47].

## 3. Results and Discussion

### 3.1. pH of Soaking Solution and Leachate

**Figure 3:**
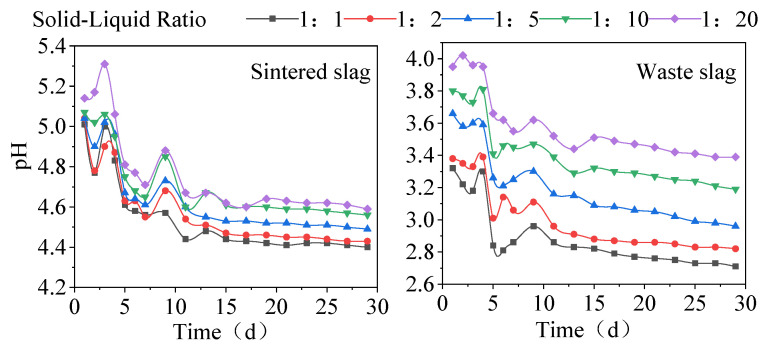
Changes in pH of the two slag soaking solutions with time.

The changes in the pH values of the solutions in the soaking tests over time are shown in Figure 3, which indicates that the pH decreased as the soaking time increased. According to Figure 3, the changes in the pH values can be divided into three stages: the early stage of soaking, the middle stage of soaking, and the late stage of soaking. In the early stage, during days 0–7, the pH of the soaking solution decreased rapidly. Under different solid:liquid ratios, the pH of the sintered slag soaking solution decreased rapidly from the initial value of 7.5 to 4.6–4.8, and the corresponding range for the waste slag decreased to 2.7–3.5. In the middle stage during days 7–15, the pH increased initially and then decreased. In the final stage, during days 15–30, the pH decreased slowly and then tended to stabilize. Under different solid:liquid ratios, the differences in the pH were smaller in the sintered slag soaking solution (the final pH ranged from 4.4 to 4.59) than in the waste slag soaking solution (the final pH ranged from 2.7 to 3.4). These results demonstrate that the slag contained acidic minerals and had a great potential for generating acid under soaking conditions. The pH of the soaking solution was negatively correlated with the solid:liquid ratio. In the present study, the water used in the soaking tests had a fixed weight of 180 g. The pH decreased as the solid:liquid ratio increased, probably due to the increase in acidic minerals at higher proportions of solid slag. The potential for generating acid was greater for the waste slag than the sintered slag, probably due to the different properties of the slag samples. According to Table 1, the sulfur content was greater in the waste slag than the sintered slag, and the sulfur content affected the pH of minerals and the potential to generate acid to a certain extent [38,48,49].

**Figure 4:**
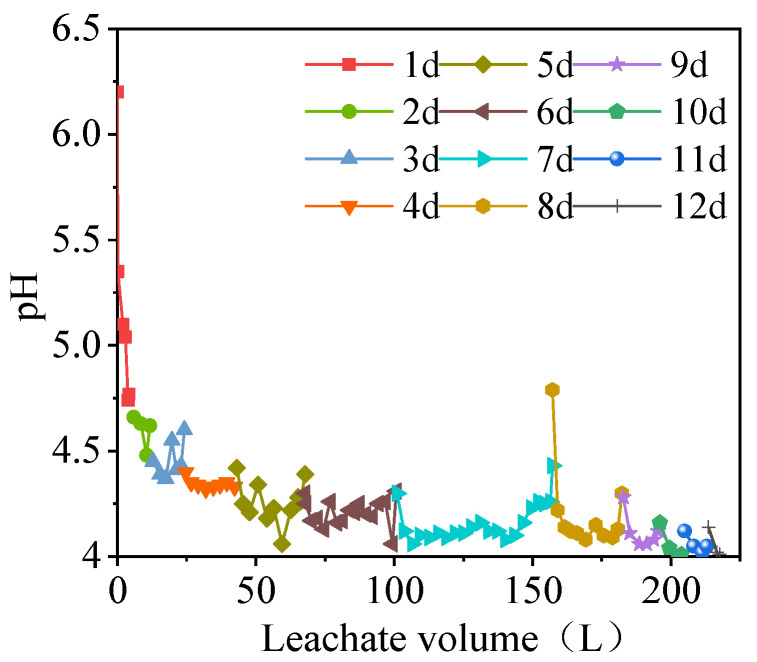
Changes in pH with leachate volume under rainfall leaching conditions.

The changes in the pH with the volume of the leachate under rainfall leaching conditions are shown in Figure 4. The pH of the leachate decreased as the leachate volume increased. The pH of the leachate decreased rapidly in the initial stage (where the pH decreased from the initial value of 6.2 to about 4.3 on the 5th day of leaching), and the rate of decrease gradually slowed in the later stage (the pH decreased from 4.3 to 4). The daily pH of the leachate also decreased rapidly initially (i.e., the pH decreased from 6.2 to 4.77 on the 1st day), before then fluctuating slightly. The pH dropped to about 4 and then stabilized. Compared with the solution in the soaking process, the leachate had a lower pH in the rainfall leaching process.

In the alum tailings soaking solution and leachate solution, the pH ranged between 2.7 and 5.3, thereby indicating that the alum tailings produced acid mine drainage under leaching conditions, and thus long-term exposure and accumulation will cause damage to the soil, surface water, and groundwater environments. The water and soil environments in the mining area are acidic, and the levels of As, Cu, Cd, Cr, Ni, Mn, and other PTMs exceed the standards [36,37,39]. The pH values in the soaking solution and leachate tended to decrease rapidly initially before then decreasing slowly, which has been due to the hydrolysis of KAl(SO_4_)_2_·12H_2_O in the alum ore generating hydroxide colloids and releasing large numbers of hydrogen ions with a rapid decrease in the pH. The reaction can be described by Equations (5) and (6) [50]. In addition, alkaline substances present in the slag consumed some H+ ions [38]. The exchangeable fraction of PTMs in the slag was completely released over the reaction period, and the carbonate-bound fraction reacted with H+ to increase the pH. The changes in the pH of the leachate in this study are consistent with the results obtained by Li et al. [51], but different from those reported by Qian et al. [52], probably due to differences in the slag compositions, i.e., acidic or alkaline.

(6) $$KAl{\left(SO_{4}\right)}_{2}=K^{+}+Al^{3+}+2SO_{4}{}^{2-}$$

(7) $$Al^{3+}+3H_{2}O⇌Al{\left(OH\right)}_{3}(colloid)+3H^{+}$$

### 3.2. Leaching Characteristics of PTMs

**Figure 5:**
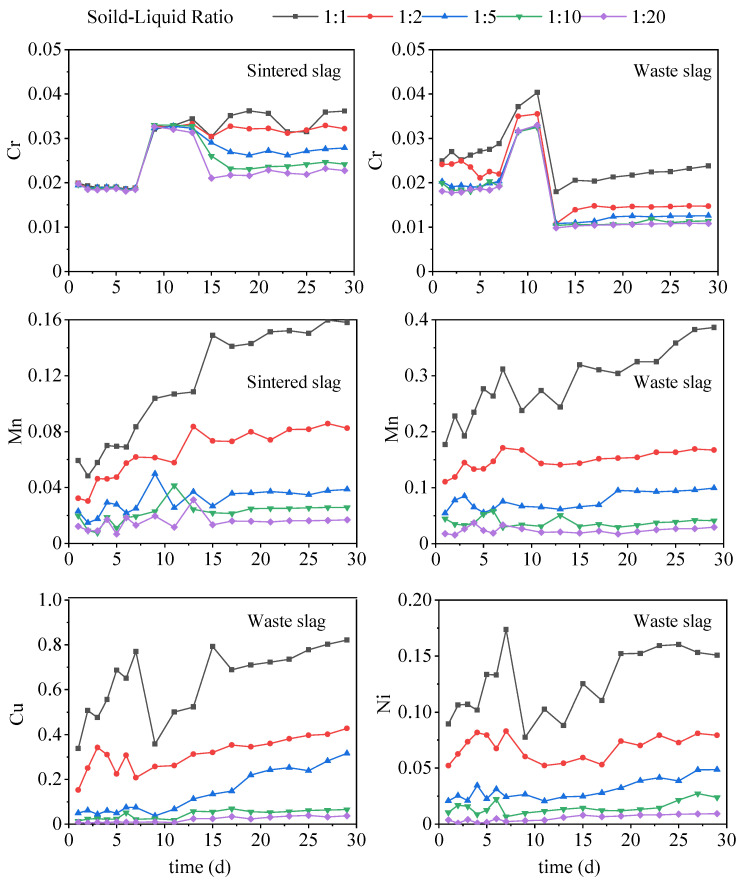
Variations in concentration of PTMs (mg/L) over time under soaking conditions.

In soaking tests, Cd was lower than the detection limit and was not detected, and Cu and Ni in the sintered slag soaking solution were also lower than the detection limits. The concentration variations of Cr and Mn in the two kinds of slag and Cu and Ni in waste slag with time under soaking conditions were shown in Figure 5. Figure 5 shows that the PTM (Cr, Cu, Mn, and Ni) concentrations all increased as the solid:liquid ratio increased. According to previous studies [53], the dominant effect of the solid:liquid ratio on PTMs release from solid waste is based on solubility. Under the fixed weight of water, the fraction of solids that can dissolve PTMs increases as the solid:liquid ratio increases, leading to a continuous increase in the release of PTMs. This should remain relatively stable when the dissolution limit is reached and will no longer increase with increases in the solid-to-liquid ratio.

The changes in the Cr ion concentrations in the two slag-soaking solutions followed a similar trend with the soaking time, where the change was relatively small during days 0–7. The concentration of Cr in the soaking solution with different solid:liquid ratios for the sintered slag was about 0.019 mg/L, and the concentration of Cr in the waste slag soaking solution varied greatly with different solid:liquid ratios, where the range was about 0.019–0.029 mg/L. During days 7–15, the Cr concentration tended to increase rapidly initially, before then decreasing rapidly, where the Cr concentration peaks with different solid:liquid ratios for the waste slag ranged from 0.032 to 0.04 mg/L, and the lowest Cr concentrations ranged from 0.011 to 0.019 mg/L. After 15 days, the changes were not significant, and the Cr concentration tended to stabilize. The peak concentration of Cr ions occurred in the middle stage of soaking, thereby indicating that the chemical fractions of Cr in the two slag types mainly comprised the oxidizable fraction or organic-bound fraction. However, the Cr concentration in the sintered slag-soaking solution was higher than the initial concentration when it was stable in the later soaking stage, whereas the Cr concentration in the waste slag-soaking solution was lower than the initial concentration when it was stable in the later stage, probably due to the lower pH of the waste slag-soaking solution. Fonseca et al. [54] showed that the capacity of soil for adsorbing Cr ions increased as the pH value decreased. The pH of the waste slag soaking solution was low in the later soaking stage (Figure 3), and thus Cr might have been re-adsorbed by the slag, thereby reducing the Cr concentration in the soaking solution. Finally, the Cr ion concentration in the sintered slag soaking solution was higher than that in the waste slag at the same solid:liquid ratio due to the difference in the Cr contents of the two types of slag (Table 1).

The Mn concentration increased with time in the early soaking stage (i.e., the Mn concentration in the soaking solution with different solid:liquid ratios increased from 0 to the range of 0.016–0.15 mg/L) but changed little with time after 15 days because a large amount of soluble Mn was precipitated out of the slag in the early soaking stage. As the soaking time increased, the proportion of soluble Mn in the slag gradually decreased, and the amount of precipitation decreased. The Mn content in the sintered slag was more than ten times that in the waste slag (Table 1), but the concentration of Mn in the sintered slag soaking solution was lower than that in the waste slag (the range of the final Mn concentration was 0.018–0.158 mg/L and 0.03–0.386 mg/L, respectively), probably due to differences in the chemical fractions of Mn in the slag. High-temperature processing and other treatments of sintered slag will lead to changes in the fractions of PTMs, thereby resulting in differences in the dissolved Mn concentration. The dissolution characteristics of Cu and Ni were similar, where the concentrations of Cu and Ni in the solution increased rapidly in the early stage (the concentrations of Cu and Ni almost reached the maximum values on the 7th day, i.e., 0.792 mg/L and 0.174 mg/L, respectively; Figure 5), before then decreasing rapidly (i.e., 0.352 mg/L and 0.075 mg/L on the 9th day, respectively), and then increasing slowly in the late stage, thereby indicating that the chemical fractions of Cu and Ni were similar in the waste slag.

**Figure 6:**
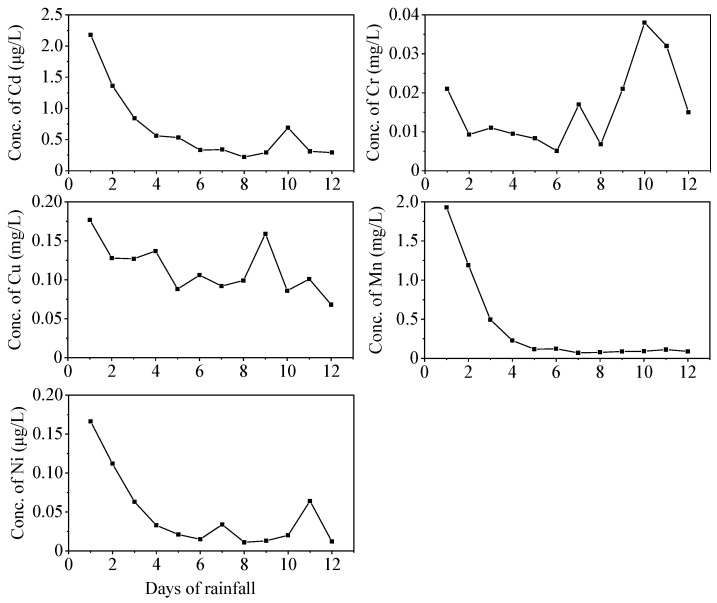
Changes in PTM concentrations in leachate.

Figure 6 shows the changes in the concentrations of PTMs in the sintered slag leachate under rainfall leaching conditions. The concentration of Cd reached a peak on the first day of leaching before then decreasing rapidly (from 2.2 to 0.38 μg/L). In the late leaching stage, the concentration peaks occurred intermittently and were smaller than those on the first day, thereby indicating that part of the chemical fractions of Cd in the sintered slag were relatively stable, where they were activated and transformed into the exchangeable fraction constantly under the repeated weathering and oxidation conditions during the rainfall leaching process. The release of Cr and Cu followed an intermittent process, where concentration peaks appeared in the early, middle, and late leaching stages (i.e., Cr concentration peaked on days 1, 7, and 10), thereby indicating that some of the chemical fractions of Cr and Cu were relatively stable in the sintered slag. The soluble fraction was generated constantly in the leaching process, but the soluble fraction of Cu gradually decreased whereas the soluble fraction of Cr tended to increase. The Mn concentration peaked (1.93 mg/L) on the first day of leaching but then decreased rapidly to a low level (0.1 mg/L) and tended to stabilize. Cr may have existed mainly in Fe-Mn oxide-bound or organic-bound fractions, which are not readily released under general conditions, whereas Mn may have existed mainly in the exchangeable fraction. The leaching characteristics of Ni were similar to those of Cd, which indicates that the chemical fractions of Ni and Cd were similar in the sintered slag.

The rapid increases in the PTM concentrations that appeared in the early stages of both the soaking process and the rainfall leaching process were probably due to the fast dissolution of the water-soluble fractions and weakly adsorbed fractions on the slag’s surface [15,55]. Due to the dissolution of the exchangeable fraction, the slag surface was degraded and the internal parts of the slag were exposed, thereby yielding adsorption sites, and some of the dissolved PTMs were re-adsorbed to decrease the concentrations of PTMs. In the late soaking stage, as the internal parts of the slag gradually reacted with the soaking solution, the fractions that were difficult to exchange were released through further oxidation [56], which was relatively slow. Accordingly, the PTM concentrations increased slowly in the late stages of soaking or leaching.

The Ni, Cu, and Cd contents in the sintered slag solution under soaking conditions were all lower than the detection limit, whereas all three metals exhibited a certain amount of dissolution under the rainfall leaching conditions. These results indicate that intermittent reoxygenation during the dynamic leaching process made the slag undergo repeated weathering and oxidation. The chemical fractions of PTMs were transformed and recombined, and the residual fraction could be continuously activated and transformed into exchangeable fractions, which were released into the leachate. By contrast, under the static soaking conditions, the slag was in the water in a state of anoxic reduction, and thus the activation rates of some internal elements in the slag were greatly reduced [57]. Previously, Huang et al. [55] and Jiang et al. [28] showed that the dissolution of PTMs was related to the elemental content, chemical fractions, adsorption capacity of slag, and solid–liquid interactions.

Except for Cr, the released contents of Cd, Cu, Mn, and Ni followed the opposite trend to the pH values of the leaching solutions, where the pH of the solution decreased but the dissolution of PTMs increased, which is consistent with the results obtained by Wang et al. and Zhang et al. [49,58]. Low pH can actually weaken the strength of metal association and reduce the negative surface charge of organic matter and Fe-Mn-Al oxides [59], thereby causing the adsorbed PTMs to desorb into the leaching solutions and an increase in the released contents of PTMs in the leaching solutions.

### 3.3. Kinetic Fitting of Cumulative PTM Release

**Figure 7:**
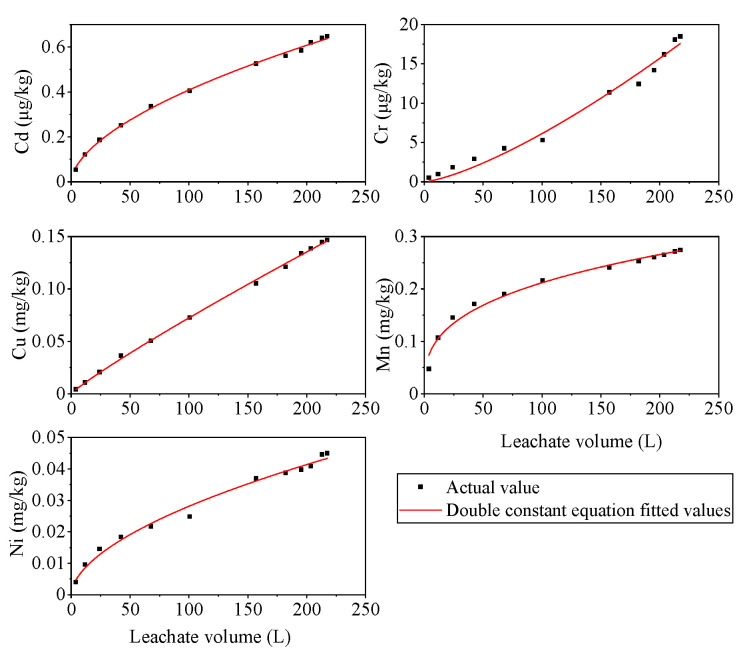
Changes in the cumulative release of Cd, Cr, Cu, Mn, and Ni from sintered slag with the leachate volume and double-constant equation fitting.

The cumulative release of Cd, Cr, Cu, Mn, and Ni into the sintered slag leachate followed an increasing trend as the leachate volume increased under simulated rainfall leaching conditions (Figure 7). Except for Cr, the increases in the cumulative release of other PTMs could be roughly divided into two stages: the rapid dissolution stage and the slow dissolution stage. In the first stage, the ionic PTMs adsorbed on the surface of the slag dissolved rapidly, and the water-soluble fraction also dissolved quickly into the leachate. In the second stage, the rate of increase in the cumulative release of Cd, Cu, Mn, and Ni gradually slowed down when the leachate volume exceeded 50 L, probably because the dissolution of the water-soluble fraction on the surface of the slag destroyed the surfaces of the metal compounds and more PTMs were released. In addition, the inner parts of the slag were then in contact with the rainfall and air, which further oxidized and released PTMs that were more difficult to exchange. The release rate of PTMs was relatively slow in this process. These results are consistent with the leaching test results obtained by Zhang et al. [60] and Li et al. [61]. Our analysis suggested that the chemical fractions of Cr in the slag mainly comprised the inactive fraction, i.e., the oxidation state or organic state, and the repeated weathering and oxidation during the rainfall leaching process promoted the transformation of Cr into the active fraction. Therefore, the increase in the cumulative release of Cr continued during the rainfall leaching period in this study.

These findings demonstrate that the leaching and release of PTMs from the tailings were relatively complex processes. A numerical model was applied to further explore the relationships between rainfall leaching and the migration and release of PTMs from alum slag, which are important for evaluating and controlling heavy metal pollution, conducting ecological risk assessments, and understanding PTM migration and transformation mechanisms. According to the results obtained in the rainfall leaching experiment, four commonly used kinetic equations comprising the first-order kinetic equation, modified Elovich equation, double constant equation, and parabola equation were used to model the relationships between the cumulative release of Cd, Cr, Cu, Mn, and Ni from sintered slag and the cumulative leachate volume. The fitting results for each model are shown in Tables S2–S5. The kinetic characteristics of the cumulative release of the PTMs were best fitted by the double constant equation (R^2^ > 0.98 for all fits; Figure 7). The first-order kinetic equation is suitable for describing a process controlled by a single diffusion mechanism, whereas the modified Elovich equation is suitable for describing a process involving a series of reaction mechanisms [62], the double constant equation is suitable for describing the heterogeneity of an energy distribution, and the parabolic equation is suitable for describing a process controlled by multiple diffusion mechanisms [61].

In the modified Elovich equation and double constant equation, parameter a represents the diffusion rate of PTMs from the solid phase to the liquid phase [55], which reveals that the diffusion rates of Mn and Ni are much higher than the other PTMs. The fitting results from the parabolic model indicate that intra-particle diffusion is the rate-limiting step in the leaching processes, especially for Mn and Cu. The fitting results showed that the dissolution and diffusion mechanism for Cr in sintered slag was relatively simple, but the Mn leaching and release process was a complex reaction process with large changes in the activation energy. The Cu release kinetic process was more complex, with greater energy inhomogeneity. Both the double constant rate equation and parabolic equation were suitable for describing the Ni and Cd release processes, thereby indicating that their leaching and release were mainly characterized by the release of different energy points and multiple diffusion mechanisms. Therefore, these kinetic equations suggest that the release of PTMs from mine tailings under rainfall leaching conditions was a complex process controlled by multiple factors.

In general, the pathways of PTMs releasing from mine tailings contain the direct dissolution of PTMs in rainfall, desorption from tailing particles, and ions exchanging with cations [63], which can be influenced by many factors, such as the reaction rate, adsorption-desorption, mineralogy of alum tailings, the acidity and alkalinity of precipitation, and the diffusion factor [15,64]. For the factors of adsorption and desorption characteristics, iron-manganese oxides play a key role in the adsorption of PTMs on mine tailings. They will be destroyed under rainfall, which results in the adsorption sites loss accordingly [65]. This is one of the reasons that the cumulative release of PTMs increases with the increase of leachate or rainfall. In the present study, the cumulative release of PTMs in the leachate was ranked in the order of Mn (0.274 mg/kg) > Cu (0.147 mg/kg) > Ni (0.045 mg/kg) > Cr (0.018 mg/kg) > Cd (0.001 mg/kg). This shows that the contents of PTMs in leachate are in general positively correlated with their contents in alum mine tailings, as well as the chemical fractions of PTMs.

### 3.4. Effects of Leaching and Release of PTMs on the Environment

The maximum concentrations of PTMs in the static soaking solution and the dynamic leaching solution were compared with the standard values in the Chinese standards for drinking water quality, as shown in Table 3. *P_i_* and WQI were used to evaluate the effects of PTMs leaching from tailings on the water quality, as shown in Table 4. The *P_i_* evaluation results showed that the pollution degree was highest for Mn and the environmental quality indexes for Mn were large in the slag soaking solution and leaching solution (*P_i_* for Mn were 1.6, 19.3, and 3.86 in the sintered slag soaking solution, the sintered slag leaching solution, and the waste slag soaking solution, respectively). The second highest pollution degree was for Ni, and the environmental quality indexes were large for Ni in both the sintered slag leaching solution and waste slag soaking solution, where the *P_i_* values were 8.3 and 8.7, respectively. The WQI evaluation results showed that the water quality was good for the sintered slag soaking solution (WQI = 67.078), whereas the water quality was the worst and undrinkable for the sintered slag leachate (WQI = 616.411), and the water quality was poor for the waste slag soaking solution (WQI = 186.9). These findings indicate that the water quality evaluation results would be different but more objective by considering the toxicity of PTMs and their effects on human health. In addition, it should be noted that according to our results, the concentration of PTMs that dissolved from waste slag was greater than that from sintered slag (i.e., the maximum concentrations of Cr dissolved from waste slag and sintered slag during the soaking process were 0.04 mg/L and 0.036 mg/L, respectively, and the data for Mn were 0.386 mg/L and 0.158 mg/L, respectively), and the PTMs were more likely to dissolve under rainfall leaching conditions than static soaking conditions, which can be confirmed by comparing the concentration of PTMs dissolved from sintered slag under the two test conditions. Thus, we suggest that the concentrations of these harmful PTMs dissolved from the waste slag will be higher under dynamic leaching conditions, which are more harmful to the environment.

On the basis of the above analysis, the health risk posed by exposure to Cd, Cr, Cu, Mn, and Ni in the leaching solution through drinking water ingestion was estimated. The results of CDI and the non-carcinogenic risk of HQ for adults are given in Tables S6–S8. The total non-carcinogenic risk (HI) values in adults were greater than 1 for both the sintered slag leachate and the waste slag soaking solution, where the HI values were 3.965 and 2.342, respectively, whereas HI for the sintered slag soaking solution was below 1. These results indicate that there is a high chronic health risk when the sintered slag leachate and waste slag soaking solution are used as drinking water, which is consistent with the water quality index (WQI) results. For the sintered slag leachate, only HQ for Cd (1.994) >1, and HQ values show that Cd and Cr make up 50.29% and 15.93%, respectively, of the total non-carcinogenic risk (HI). For the waste slag soaking solution, Cd was not detected, and Cr makes up 28.38% of the total non-carcinogenic risk (HI). These results indicate a high non-carcinogenic risk of exposure to Cd and Cr in the leaching solution used for drinking purposes. In addition to causing adverse non-carcinogenic health effects, Cd and Cr are categorized as carcinogenic substances [66]. Previous studies showed that HQ values were usually higher for children than for adults [45,46,67]. One of the main reasons for the high non-carcinogenic risk in children is their lower body weight (BW) compared to adults [68].

Studies have shown that the levels of Cd, Cr, Cu, Mn, Ni, and other harmful PTMs in soil and surface waters in alum mining areas exceed the standards [34,37], thereby demonstrating that the leaching of alum tailings has caused severe pollution in the surrounding environment. Therefore, measures need to be implemented to prevent the exposure of tailings to the air and rainwater to control the leaching of alum tailings and reduce human exposure to Cd, Cr, and other PTMs through drinking water.

## 4. Conclusions

Under soaking and rainfall leaching conditions, acid mine drainage with a low pH (roughly in the range of 2.7–4.59) and PTEs comprising Cd, Cr, Cu, Mn, and Ni were leached from both the sintered slag and waste slag. pH values of the two slag soaking solutions were negatively correlated with the solid:liquid ratio under the fixed water weight (180 g) in the present study, probably due to the increase in the acid-producing substance (KAl(SO_4_)_2_·12H_2_O) at higher proportions of solid slag. The PTMs’ leaching and release capacities were higher for waste slag than for sintered slag. The maximum concentrations of Cr dissolved from waste slag and sintered slag during the soaking process were 0.04 mg/L and 0.036 mg/L, respectively, and the data for Mn were 0.386 mg/L and 0.158 mg/L, respectively. The concentrations of PTMs (Cd, Cr, Cu, Mn, and Ni) dissolved from sintered slag were greater under rainfall leaching conditions than soaking conditions (the difference in Cr concentration was the smallest, 5.6%). The double constant equation fitted best to the kinetic characteristics of cumulative PTMs release under rainfall leaching conditions (R^2^ > 0.98 for all fits). Single factor contamination indexes *P_i_* for Mn and Ni in almost all the leaching solutions (except for Ni in the sintered slag soaking solution) exceeded 1. However, considering the biological toxicity of PTMs, the results of the WQI showed that the water quality of the sintered slag leachate was the worst and undrinkable, and the water quality of the waste slag soaking solution was poor. The total non-carcinogenic risk (HI) values for adults indicate a high chronic health risk of exposure to PTMs in the sintered slag leachate and waste slag soaking solution used for drinking purposes, as HI values exceeded the safe level of 1 for both. Among them, Cd and Cr make up 50.29% and 15.93%, respectively, of the total risk for the sintered slag leachate, with a HQ of 1.994 for Cd, and Cr makes up 28.38% of the total risk for the waste slag soaking solution. Therefore, the impacts of the acid mine drainage leached from alum mine tailings on the environment require further attention, and measures must be implemented to avoid the exposure of slag to water and air.

## Figures and Tables

**Table 1:** Content of the main elements in alum ore slag.

	Al mg/kg	Cd mg/kg	Cr mg/kg	Cu mg/kg	Mn mg/kg	Ni mg/kg	S %
Sintered slag	96,290	1.1	65.2	377.3	126	17.5	2.28
Waste slag	79,400	1.2	24.1	54.8	9.4	0.1	7.77
GB 15618-2018 Screening value		0.3	150	50		60	

**Table 2:** Rainfall distribution and rainfall duration in simulated rainfall leaching experiments.

Time (Days)	1	2	3	4	5	6	7	8	9	10	11	12
rainfall (mm)	40	60	90	120	140	240	300	145	75	50	45	35
rainfall duration (h)	2.7	4.0	6.0	8.0	9.3	16.0	20.0	9.7	5.0	3.3	3.0	2.3

**Table 3:** Comparison of the maximum dissolved concentrations of PTMs in static soaking and dynamic leaching tests with Chinese standards for drinking water quality (bold values exceed the standards).

PTM	Chinese Standards for Drinking Water Quality (GB5749-2022)	Sintered Slag		Waste Slag	Weight (w_i_)	Relative Weight (W_i_)
(mg/L)		Static Soaking	Dynamic Leaching	Static Soaking		
Cd	≤0.005	——	0.002	——	5	0.278
Cr	≤0.05	0.036	0.038	0.04	5	0.278
Cu	≤1	——	0.177	0.821	2	0.111
Mn	≤0.1	**0.158**	**1.93**	**0.386**	5	0.278
Ni	≤0.02	**0.0086**	**0.166**	**0.174**	1	0.056

**Table 4:** Environmental risk assessments for pollution by PTMs in leaching solutions (bold values are single-factor contamination indexes).

PTM		Cd	Cr	Cu	Mn	Ni	WQI	Result
Sintered slag	Static soaking	--	**0.720**	**0.022**	**1.600**	**0.430**	67.078	Good
	Dynamic leaching	**0.400**	**0.760**	**0.177**	**19.300**	**8.300**	616.411	Undrinkable
Waste slag	Static soaking	--	**0.800**	**0.821**	**3.860**	**8.700**	186.900	Poor

## Data Availability

Not applicable.

## Supplementary Materials

The following supporting information can be downloaded at: https://www.mdpi.com/article/10.3390/ijerph192417063/s1. Table S1: The chemical analysis results of main elements in alum ore slag, Table S2: Fitting results of cumulative heavy metal release by First-order kinetic equation, Table S3: Fitting results of cumulative heavy metal release by Modified Elovich equation, Table S4: Fitting results of cumulative heavy metal release by Double constant equation, Table S5: Fitting results of cumulative heavy metal release by Parabola equation, Table S6: CDI and non-carcinogenic risk of HQ for PTEs from the sintered slag leachate used for drinking purpose, Table S7: CDI and non-carcinogenic risk of HQ for PTEs from the sintered slag soaking solution used for drinking purpose, Table S8: CDI and non-carcinogenic risk of HQ for PTEs from the waste slag soaking solution used for drinking purpose

## References

[1] Wang Y., Wang R., Fan L., Chen T., Bai Y., Yu Q., Liu Y. (2017). Assessment of multiple exposure to chemical elements and health risks among residents near Huodehong lead-zinc mining area in Yunnan, Southwest China. Chemosphere.

[2] Shi M., Min X., Ke Y., Lin Z., Yang Z., Wang S., Peng N., Yan X., Luo S., Wu J. (2021). Recent progress in understanding the mechanism of heavy metals retention by iron (oxyhydr) oxides. Sci. Total Environ..

[3] Menzel K., Barros L., García A., Ruby-Figueroa R., Estay H. (2021). Metal sulfide precipitation coupled with membrane filtration process for recovering copper from acid mine drainage. Sep. Purif. Technol..

[4] Khoeurn K., Sakaguchi A., Tomiyama S., Igarashi T. (2019). Long-term acid generation and heavy metal leaching from the tailings of Shimokawa mine, Hokkaido, Japan: Column study under natural condition. J. Geochem. Explor..

[5] Sun R., Yang J., Xia P., Wu S., Lin T., Yi Y. (2020). Contamination features and ecological risks of heavy metals in the farmland along shoreline of Caohai plateau wetland, China. Chemosphere.

[6] Parviainen A. (2009). Tailings Mineralogy and Geochemistry at the Abandoned Haveri Au-Cu Mine, SW Finland. Mine Water Environ..

[7] Tabelin C.B., Uyama A., Tomiyama S., Villacorte-Tabelin M., Phengsaart T., Silwamba M., Jeon S., Park I., Arima T., Igarashi T. (2022). Geochemical audit of a historical tailings storage facility in Japan: Acid mine drainage formation, zinc migration and mitigation strategies. J. Hazard Mater.

[8] Zhu G., Xiao H., Guo Q., Song B., Zheng G., Zhang Z., Zhao J., Okoli C.P. (2018). Heavy metal contents and enrichment characteristics of dominant plants in wasteland of the downstream of a lead-zinc mining area in Guangxi, Southwest China. Ecotoxicol. Environ. Saf..

[9] Dong L., Deng S., Wang F. (2020). Some developments and new insights for environmental sustainability and disaster control of tailings dam. J. Clean. Prod..

[10] Rana N.M., Ghahramani N., Evans S.G., McDougall S., Small A., Take W.A. (2021). Catastrophic mass flows resulting from tailings impoundment failures. Eng. Geol..

[11] Ministry of Ecology and Environment of the People’s Republic of China (2020). Annual Report on the Prevention and Control of Environmental Pollution by Solid Wastes in Large and Medium Cities in 2020.

[12] Sun Z., Xie X., Wang P., Hu Y., Cheng H. (2018). Heavy metal pollution caused by small-scale metal ore mining activities: A case study from a polymetallic mine in South China. Sci. Total Environ..

[13] Qin H.B., Takeichi Y., Nitani H., Terada Y., Takahashi Y. (2017). Tellurium Distribution and Speciation in Contaminated Soils from Abandoned Mine Tailings: Comparison with Selenium. Environ. Sci. Technol..

[14] Lindsay M.B.J., Moncur M.C., Bain J.G., Jambor J.L., Ptacek C.J., Blowes D.W. (2015). Geochemical and mineralogical aspects of sulfide mine tailings. Appl. Geochem..

[15] Sun R., Gao Y., Yang Y. (2022). Leaching of heavy metals from lead-zinc mine tailings and the subsequent migration and transformation characteristics in paddy soil. Chemosphere.

[16] Assawincharoenkij T., Hauzenberger C., Sutthirat C. (2017). Mineralogy and geochemistry of tailings from a gold mine in northeastern Thailand. Hum. Ecol. Risk Assess..

[17] Tabelin C.B., Corpuz R.D., Igarashi T., Villacorte-Tabelin M., Alorro R.D., Yoo K., Raval S., Ito M., Hiroyoshi N. (2020). Acid mine drainage formation and arsenic mobility under strongly acidic conditions: Importance of soluble phases, iron oxyhydroxides/oxides and nature of oxidation layer on pyrite. J. Hazard Mater.

[18] Chen M., Lu G., Wu J., Sun J., Yang C., Xie Y., Wang K., Deng F., Yi X., Dang Z. (2020). Acidity and metallic elements release from AMD-affected river sediments: Effect of AMD standstill and dilution. Environ. Res..

[19] Bhowmick S., Pramanik S., Singh P., Mondal P., Chatterjee D., Nriagu J. (2018). Arsenic in groundwater of West Bengal, India: A review of human health risks and assessment of possible intervention options. Sci. Total Environ..

[20] Desogus P., Manca P.P., Orru G., Zucca A. (2013). Stabilization-solidification treatment of mine tailings using Portland cement, potassium dihydrogen phosphate and ferric chloride hexahydrate. Miner. Eng..

[21] Imoto Y., Yasutaka T., Someya M., Higashino K. (2018). Influence of solid-liquid separation method parameters employed in soil leaching tests on apparent metal concentration. Sci. Total Environ..

[22] Hage J.L., Mulder E. (2004). Preliminary assessment of three new European leaching tests. Waste Manag..

[23] Kumar M., Furumai H., Kurisu F., Kasuga I. (2013). Potential mobility of heavy metals through coupled application of sequential extraction and isotopic exchange: Comparison of leaching tests applied to soil and soakaway sediment. Chemosphere.

[24] Little K.W., Koralegedara N.H., Northeim C.M., Al-Abed S.R. (2017). Decision support for environmental management of industrial non-hazardous secondary materials: New analytical methods combined with simulation and optimization modeling. J. Environ. Manag..

[25] Wang P., Sun Z., Hu Y., Cheng H. (2019). Leaching of heavy metals from abandoned mine tailings brought by precipitation and the associated environmental impact. Sci. Total Environ..

[26] Geng H., Wang F., Yan C., Tian Z., Chen H., Zhou B., Yuan R., Yao J. (2020). Leaching behavior of metals from iron tailings under varying pH and low-molecular-weight organic acids. J. Hazard Mater..

[27] Akhavan A., Golchin A. (2021). Estimation of arsenic leaching from Zn-Pb mine tailings under environmental conditions. J. Clean. Prod..

[28] Jiang L., Sun H., Peng T., Ding W., Liu B., Liu Q. (2021). Comprehensive evaluation of environmental availability, pollution level and leaching heavy metals behavior in non-ferrous metal tailings. J. Environ. Manag..

[29] Liu B., Peng T., Sun H., Yue H. (2017). Release behavior of uranium in uranium mill tailings under environmental conditions. J. Environ. Radioact..

[30] Santos A.L.A., Becheleni E.M.A., Viana P.R.M., Papini R.M., Silvas F.P.C., Rocha S.D.F. (2021). Kinetics of Atmospheric Leaching from a Brazilian Nickel Laterite Ore Allied to Redox Potential Control. Min. Met. Explor..

[31] Zuo D., Huang J., Yue M., Liu S. (2019). Morphological characteristics and ecological risk assessment of soil heavy metals in Lujiang alumite mine. Ecol. Sci..

[32] Li X.X., Zhou T.F., White N.C., Fan Y., Zhang L.J., Xie J., Liu Y.N., Xiao X. (2020). Formation of the Fanshan lithocap and implications for exploration in the Luzong Basin, Anhui Province, China. Ore Geol. Rev..

[33] Wang L.Y., Xue N.N., Zhang Y.M., Hu P.C. (2021). Controlled Hydrothermal Precipitation of Alunite and Natroalunite in High-Aluminum Vanadium-Bearing Aqueous System. Minerals.

[34] Zhu H., Ren J., Yang Q., Zhou B., Jia Y., Lv J., Zhou X., Nie W. (2021). Sources Analysis of Acid Drainage From Lujiang Alunite Mine Based on Stable Isotope Composition of Hydrogen and Oxygen. Resour. Environ. Yangtze Basin.

[35] Qian L. (2018). Study on the Dynamic Simulation and Control of Acid Water from Alunite Mine in Lujiang Country. Master’s Thesis.

[36] Wang C. (2018). Lujiang Iron Ore Mine Acid Water Formation Mechanism and Dynamic Simulation of Water Quality. Master’s Thesis.

[37] Zhu L. (2020). Study on Comprehensive Evaluation and Governance System of Environmental Quality in Typical Alunite MiningArea. Master’s Thesis.

[38] Huang J., Zuo D., Yue M. (2017). Assessing the acid generation potential of Lujiang alum ore. J. Anqing Norm. Univ. (Nat. Sci. Ed.).

[39] Zhou B., Guo J., Chen X., Yang Q., Zhu H., Duan M., Li X., Zhou D., Yang Y. (2021). Source apportionment of soil heavy metals in abandoned mining areas in Dafan Mountain of Anhui Province based on the UNMIX model. Trans. Chin. Soc. Agric. Eng..

[40] Ministry of Ecology and Environment (MEE) (2018). Soil Environmental Quality Risk ControlStandard for Soil Contamination of Agricultural Land (GB 15618-2018).

[41] State Administration for Market Regulation, Standardization Administration of China (2022). Standards for Drinking Water Quality (GB5749–2022).

[42] Xiao J., Wang L.Q., Deng L., Jin Z.D. (2019). Characteristics, sources, water quality and health risk assessment of trace elements in river water and well water in the Chinese Loess Plateau. Sci. Total Environ..

[43] Khandare A.L., Validandi V., Rajendran A., Singh T.G., Thingnganing L., Kurella S., Nagaraju R., Dheeravath S., Vaddi N., Kommu S. (2020). Health risk assessment of heavy metals and strontium in groundwater used for drinking and cooking in 58 villages of Prakasam district, Andhra Pradesh, India. Environ. Geochem. Health.

[44] (2020). USEPA Integrated Risk Information System (IRIS). http://www.epa.gov/iris/.

[45] Joodavi A., Aghlmand R., Podgorski J., Dehbandi R., Abbasi A. (2021). Characterization, geostatistical modeling and health risk assessment of potentially toxic elements in groundwater resources of northeastern Iran. J. Hydrol.-Reg. Stu..

[46] Eslami H., Esmaeili A., Razaeian M., Salari M., Hosseini A.N., Mobini M., Barani A. (2022). Potentially toxic metal concentration, spatial distribution, and health risk assessment in drinking groundwater resources of southeast Iran. GeoSci. Front..

[47] Pecina V., Brtnicky M., Baltazar T., Juricka D., Kynicky J., Galiova M.V. (2021). Human health and ecological risk assessment of trace elements in urban soils of 101 cities in China: A meta-analysis. Chemosphere.

[48] Embile R.F., Walder I.F., Mahoney J.J. (2018). Forsterite and pyrrhotite dissolution rates in a tailings deposit obtained from column leaching experiments and inverse modeling: A novel method for a mine tailings sample. Appl. Geochem..

[49] Wang G.F., Xiao H.Z., Liang G.C., Zhu J.L., He C.L., Ma S.J., Shuai Z., Komarneni S. (2022). Leaching characteristics and stabilization of heavy metals in tin-polymetallic tailings by sodium diethyl dithiocarbamate intercalated montmorillonite (DDTC-Mt). J. Clean. Prod..

[50] Berger A.C., Bethke C.M., Krumhansl J.l. (2000). A process model of natural attenuation in drainage from a historic mining district. Appl. Geochem..

[51] Li Y., Wu P., Xing N., Dang Z., Yi X., Luo H. (2010). Simulated Weathering and Leaching Experiments on Sulphide-Rich Tailings at Dabaoshan Deposit. Acta Min. Sin..

[52] Qian L., Li B., Chen X., Li X., Lin H. (2020). Leaching characteristics and release rule of heavy metals from gold tailings. J. Southeast Univ. Nat. Sci. Ed..

[53] Huang Y.M., Liu J.L., Wang G., Bi X.Y., Sun G.Y., Wu X., Wang Q.F., Li Z.G. (2022). Concentrations, Speciation, and Potential Release of Hazardous Heavy Metals from the Solid Combustion Residues of Coal-Fired Power Plants. Int. J. Environ. Res. Public Health.

[54] Fonseca B., Teixeira A., Figueiredo H., Tavares T. (2009). Modelling of the Cr(VI) transport in typical soils of the North of Portugal. J. Hazard Mater.

[55] Huang Z., Jiang L., Wu P., Dang Z., Zhu N., Liu Z., Luo H. (2020). Leaching characteristics of heavy metals in tailings and their simultaneous immobilization with triethylenetetramine functioned montmorillonite (TETA-Mt) against simulated acid rain. Environ. Pollut..

[56] Zhou S., Wang Y., Deng X., Deng R., Wang J., Ren B. (2022). Release law of Sb, As, and Hg in antimony mine wastes under simulated acid rain. J. Civ. Environ. Eng..

[57] Li J., Chen D., Wu H., Wu W., Chen N. (2016). Release and Migration Behavior of Metals Such As Thallium from Pyrite Tailings under the Condition of Water Seal. Ecol. Environ. Sci..

[58] Zhang S., He X., Li Y., Fang Z., Wang H. (2018). Leaching experimental study on heavy metals in soil lead-zinc mine. J. Min. Sci. Technol..

[59] Du Laing G., Rinklebe J., Vandecasteele B., Meers E., Tack F.M.G. (2009). Trace metal behaviour in estuarine and riverine floodplain soils and sediments: A review. Sci. Total Environ..

[60] Zhang Y., Ren B.Z., Hursthouse A., Deng R.J., Hou B.L. (2019). Leaching and Releasing Characteristics and Regularities of Sb and As from Antimony Mining Waste Rocks. Polish J. Environ. Stud..

[61] Li S., Fang B., Wang D., Wang X., Man X., Zhang X. (2019). Leaching Characteristics of Heavy Metals and Plant Nutrients in the Sewage Sludge Immobilized by Composite Phosphorus-Bearing Materials. Int. J. Environ. Res. Public Health.

[62] Inyang H.I., Onwawoma A., Bae S. (2016). The Elovich equation as a predictor of lead and cadmium sorption rates on contaminant barrier minerals. Soil. Tillage Res..

[63] Huang L.M., Jin Q., Tandon P., Li A.M., Shan A.D., Du J.J. (2018). High-resolution insight into the competitive adsorption of heavy metals on natural sediment by site energy distribution. Chemosphere.

[64] Alghanmi Shorouq I., Al Sulami Amani F., El-Zayat Tahani A., Alhogbi Basma G., Salam Mohamed A. (2015). Acid leaching of heavy metals from contaminated soil collected from Jeddah, Saudi Arabia: Kinetic and thermodynamics studies. Int. Soil Water Conserv. Res..

[65] Clozel B., Ruban V., Durand C., Conil P. (2006). Origin and mobility of heavy metals in contaminated sediments from retention and infiltration ponds. Appl. Geochem..

[66] World Health Organization-WHO (2017). Guidelines for Drinking-Water Quality: Fourth Edition Incorporating the First Addendum.

[67] Malakootian M., Mohammadi A., Faraji M. (2020). Investigation of physicochemical parameters in drinking water resources and health risk assessment: A case study in NW Iran. Environ. Earth Sci..

[68] Fallahzadeh R.A., Khosravi R., Dehdashti B., Ghahramani E., Omidi F., Adli A., Miri M. (2018). Spatial distribution variation and probabilistic risk assessment of exposure to chromium in ground water supplies; a case study in the east of Iran. Food Chem. Toxicol..

