# Supplementary material for: Leaching Characteristics of Potentially Toxic Metals from Tailings at Lujiang Alum Mine, China
Source: Int J Environ Res Public Health. 2022 Dec 19;19(24):17063. doi: 10.3390/ijerph192417063 (PMC9779374; doi:10.3390/ijerph192417063)
Supplement: Supplementary file 1 [file ijerph-19-17063-s001.zip › ijerph-1999987-supplementary.pdf]

Table S1: The chemical analysis results of main elements in alum ore slag.

	SiO_2 %	Al_2O_3 %	Fe_2O_3 %	CaO %	MgO %	K_2O %	Na_2O %	MnO_2 %	P_2O_5 %	TiO_2 %
Sintered slag	81.45	8.98	6.45	0.66	0.58	0.99	0.086	0.344	0.1896	0.269
Waste slag	93.51	2.34	1.26	0.22	0.58	1.5	0.1	0.028	0.095	0.359

Table S2: Fitting results of cumulative heavy metal release by First-order kinetic equation.

	$lny = a + bx$				
Element	a	b	ME	RMSE	R^2
Cd	-8.64±0.135	0.0062±7.21E-4	3.41E-06	5.57E-05	0.919
Cr	-6.35±0.132	0.109±6.63E-4	1.03E-04	7.42E-04	0.986
Cu	-3.716±0.164	0.0085±8.41E-4	1.32E-03	1.07E-02	0.955
Mn	-2.1±0.113	0.0039±6.37E-4	6.06E-04	2.87E-02	0.815
Ni	-4.404±0.119	0.006±6.34E-4	1.83E-04	3.39E-03	0.934

Table S3: Fitting results of cumulative heavy metal release by Modified Elovich equation.

	$y = a \times \ln x + b$				
Element	a	b	ME	RMSE	R^2
Cd	1.56E-4±1.37E-5	-2.57E-4±6.11E-4	-1.58E-10	5.51E-05	0.922
Cr	0.0045±8.31E-4	-0.0104±3.7E-3	1.20E-06	3.34E-03	0.729
Cu	0.038±5.09E-3	-0.083±0.227	1.67E-06	2.04E-02	0.838
Mn	0.055±1.43E-3	-0.0313±6.35E-3	-8.33E-07	5.73E-03	0.992
Ni	0.104±1.02E-3	-0.016±4.56E-3	8.33E-07	4.11E-03	0.903

Table S4: Fitting results of cumulative heavy metal release by Double constant equation.

	$lny = a + b \times lnx$				
Element	a	b	ME	RMSE	R^2
Cd	2.93E-5±1.88E-6	0.572±0.125	2.55E-07	8.90E-06	0.997
Cr	1.19E-5±7.56E-6	1.356±0.121	-2.02E-04	8.37E-04	0.984
Cu	1.14E-3±1.13E-4	0.902±0.019	-1.51E-04	1.84E-03	0.998
Mn	0.047±4.36E-3	0.326±0.012	5.12E-04	9.33E-03	0.981
Ni	2.16E-3±3.30E-4	0.557±0.029	-5.67E-05	1.49E-03	0.987

Table S5: Fitting results of cumulative heavy metal release by Parabola equation.

	$y = ax^{0.5} + b$				
Element	a	b	ME	RMSE	R^2
Cd	4.56E-05±6.89E-7	-4.03E-5±7.49E-6	-1.25E-10	9.84E-06	0.997
Cr	0.0014±1.42E-4	-0.0049±1.55E-3	1.64E-06	2.03E-03	0.897
Cu	0.0117±5.69E-4	-0.0333±6.19E-3	8.33E-07	8.14E-03	0.974
Mn	0.0152±9.74E-4	0.0529±0.106	8.33E-07	1.39E-02	0.956
Ni	0.0030±1.11E-4	-0.0018±1.21E-3	-2.17E-19	1.58E-03	0.985

Table S6: CDI and non-carcinogenic risk of HQ for PTEs from the sintered slag leachate used for drinking purpose.

Element	Max Ci<br>mg/L	IR (adults)<br>L/d	BW<br>kg	CDI<br>mg/kg/day	RfD<br>mg/kg/day	HQ	HQ/HI<br>%
Cd	0.02	3.49	70	0.000997	0.0005	1.994	50.29
Cr	0.038	3.49	70	0.001895	0.003	0.632	15.926
Cu	0.177	3.49	70	0.008825	0.037	0.239	6.015
Mn	1.93	3.49	70	0.096224	0.14	0.687	17.333
Ni	0.166	3.49	70	0.008276	0.02	0.414	10.436
HI						3.965	

Table S7: CDI and non-carcinogenic risk of HQ for PTEs from the sintered slag soaking solution used for drinking purpose.

Element	Max Ci<br>mg/L	IR (adults)<br>L/d	BW<br>kg	CDI<br>mg/kg/day	RfD<br>mg/kg/day	HQ
Cd	--	3.49	70	--	0.0005	--
Cr	0.036	3.49	70	0.001795	0.003	0.598
Cu	--	3.49	70	--	0.037	--
Mn	0.158	3.49	70	0.007877	0.14	0.056
Ni	0.0086	3.49	70	0.000429	0.02	0.021
HI						0.676

Table S8: CDI and non-carcinogenic risk of HQ for PTEs from the waste slag soaking solution used for drinking purpose.

Element	Max Ci<br>mg/L	IR (adults)<br>L/d	BW<br>kg	CDI<br>mg/kg/day	RfD<br>mg/kg/day	HQ	HQ/HI<br>%
Cd	--	3.49	70	--	0.0005	--	--
Cr	0.04	3.49	70	0.001994	0.003	0.665	28.381
Cu	0.821	3.49	70	0.040933	0.037	1.106	47.231
Mn	0.386	3.49	70	0.019245	0.14	0.137	5.869
Ni	0.174	3.49	70	0.008675	0.02	0.434	18.519
HI						2.342	
